# The Protective Effects of *Lactobacillus plantarum* KLDS 1.0344 on LPS-Induced Mastitis *In Vitro* and *In Vivo*

**DOI:** 10.3389/fimmu.2021.770822

**Published:** 2021-11-09

**Authors:** Qingxue Chen, Song Wang, Jiayao Guo, Qinggang Xie, Smith Etareri Evivie, Yue Song, Bailiang Li, Guicheng Huo

**Affiliations:** ^1^ Key Laboratory of Dairy Science, Ministry of Education, Northeast Agricultural University, Harbin, China; ^2^ Food College, Northeast Agricultural University, Harbin, China; ^3^ Heilongjiang Feihe Dairy Company Ltd., Qiqihaer, China; ^4^ Department of Animal Science, Faculty of Agriculture, University of Benin, Benin City, Nigeria; ^5^ Department of Food Science and Human Nutrition, Faculty of Agriculture, University of Benin, Benin City, Nigeria

**Keywords:** *Lactobacillus plantarum*, mastitis, antibacterial, immunomodulation, signal path

## Abstract

Cow mastitis, which significantly lowers milk quality, is mainly caused by pathogenic bacteria such as *E. coli*. Previous studies have suggested that lactic acid bacteria can have antagonistic effects on pathogenic bacteria that cause mastitis. In the current study, we evaluated the *in vitro* and *in vivo* alleviative effects of *L. plantarum* KLDS 1.0344 in mastitis treatment. *In vitro* antibacterial experiments were performed using bovine mammary epithelial cell (bMEC), followed by *in vivo* studies involving mastitis mouse models. *In vitro* results indicate that lactic acid was the primary substance inhibiting the *E. coli* pathogen. Meanwhile, treatment with *L. plantarum* KLDS 1.0344 can reduce cytokines’ mRNA expression levels in the inflammatory response of bMEC induced by LPS. *In vivo*, the use of this strain reduced the secretion of inflammatory factors IL-6, IL-1β, and TNF-α, and decreased the activity of myeloperoxidase (MPO), and inhibited the secretion of p-p65 and p-IκBα. These results indicate that *L. plantarum* KLDS 1.0344 pretreatment can reduce the expression of inflammatory factors by inhibiting the activation of NF-κB signaling pathway, thus exerting prevent the occurrence of inflammation *in vivo*. Our findings show that *L. plantarum* KLDS 1.0344 has excellent properties as an alternative to antibiotics and can be developed into lactic acid bacteria preparation to prevent mastitis disease.

## Introduction

Cow mastitis is caused by pathogenic bacteria (1), which is one of the most common and harmful diseases in the livestock industry. It is highly prevalent and challenging to cure, causing a decline in milk quality and substantial economic losses (2, 3). The main pathogenic factor of mastitis in cows is infection by pathogenic microorganisms. A recent report revealed that *E. coli* was the most frequently isolated pathogen in cows with mastitis in cows farming and the primary causative agent of acute cow mastitis (4, 5). The lipopolysaccharide (LPS) in *E. coli* can be recognized by TLR4, which the primary pattern recognition receptor of innate immunity (6). LPS induces the expression of inflammatory cytokines such as TNF-α, IL-6, and IL-1β in the mammary glands, leading to mastitis in cows (7).

There is no proven vaccine to prevent and treat the disease, and it is still widely treated with many antibiotics (8, 9). The continued use of antibiotics can also cause an increase in resistance to important pathogenic bacteria (10). It brings more challenges to the prevention and treatment of diseases. Therefore, developing a novel, safe, and new clinical drug that can replace antibiotics is of great significance and value. As a probiotics, *Lactobacilli* have many beneficial health effects on humans and animals. In addition, existing studies have shown that *lactobacilli* can act as antagonists against the pathogens that cause mastitis (11, 12). Lactic acid bacteria can produce lactic acid and other antibacterial substances in the metabolic process (13), thus inhibiting the growth and reproduction of pathogenic bacteria (14).

Some studies claim that when ingested into the body, some *Lactobacilli* strains promote the production of several metabolites that can also exert anti-inflammatory effects (15) and could be a viable alternative in human mastitis treatment (14). LPS induces pro-inflammatory cytokines that trigger mastitis (16). The metabolites produced by *L. plantarumcan* reduce the levels of these pro-inflammatory factors reported by Chen et al. (17). Briefly, it is the inhibition of LPS-induced activation of NF-κB, which blocks the expression of inflammatory cytokines such as IL-1β, TNF-α, and IL-6 (18). Therefore, *L. plantarum* supplementation may be a potential strategy for mastitis patients. However, there are few studies on the mechanisms of *L. plantarum* to alleviate mastitis. In this study, we selected a potential probiotic *L. plantarum* KLDS1.0344 with a significant bacteriostatic effect to investigate the inhibition factors and explore the bactericidal activity and bactericidal mechanism. We analyzed its effect on the expression of inflammatory factors secreted by mammary epithelial cells, and whether it has a preventive effect on inflammation production in *in vivo* tests, and whether it has preventive and therapeutic implications for mastitis caused by relevant pathogenic bacteria, laying the foundation for the development of relevant probiotic preparations.

## Materials and Methods

### Bacterial Strain and Culture Conditions

The *L. plantarum* KLDS 1.0344 strain was isolated from Inner Mongolia traditional fermented dairy products and preserved at the Key Laboratory of Dairy Science, Ministry of Education of the Northeast Agricultural University (NEAU), China. *Lactobacillus* and *E. coli* were inoculated into liquid De Man, Rogosa, and Sharpe (MRS) medium, Luria-Bertani (LB) medium at a 2% inoculum; all were cultured at 37°C at a constant temperature. Bacteria were sub­cultured twice before the experiment. As previously mentioned, bMEC were cultivated in Dulbecco’s modified Eagle’s minimal essential medium(DMEM, HyClone, USA) supplemented with 10% fetal bovine serum (Gibco, USA) and 1% penicillin/streptomycin (Gibco, USA) (19). All cell cultures were maintained at 37°C under an atmosphere of 5% CO2 and 95% air.

### Preparation of the Cell-Free Fermentation Supernatant

The *L. plantarum* KLDS 1.0344 was passed to the third generation and cultured for 18 h, and the obtained fermentation broth was centrifuged at 8000 r/min for 10 minutes using a 4°C constant temperature centrifuge. The supernatant was collected and placed in a refrigerator at 4°C for temporary storage.

### Antibacterial Properties of *L. plantarum* KLDS 1.0344

The co-cultivation method was used to study the bactericidal activity of the *L. plantarum* KLDS 1.0344 on *E. coli* ATCC25922. 5mL CFS was mixed on the medium containing *E. coli* ATCC25922 (10^7^ CFU/mL) and cultured at 37°C. The saline and MRS medium without bacteria (pH=4) were used as controls, and the bacteria were counted on the plate at different times (1 h, 2 h, 3 h). The test was repeated three times.

### Preliminary Determination of Bacteriostatic Substances

The CFS were adjusted to pH 6.5 with NaOH (2 mol/L) and respectively treated by adding different components including catalase (dissolved in PBS), trypsin, proteinase K, and pepsin. The final concentration of each protease is 1 mg/mL. For the lactic acid group, adjust the pH value of the MRS medium with a lactic acid solution to be the same as that of CFS. It was then filtered and sterilized, and the CFS without any treatment was used as a control. Bacterial inhibition experiments were performed on the treated CFS using the agar diffusion method’s (20). At the same time, bacteriocin-related genes were amplified by PCR and compared with *L. plantarum* KLDS1.0344. Specific primers were designed as shown in **Figure S1**, PCR was performed for rapid amplification. These genes were present in *L. plantarum* C11, V90, J51, J23, WCFS1, NC8, and 423.

### The Influence of pH on Antibacterial Activity

Adjust the pH value of CFS to 4.5, 5.0, 5.5, or 6.0, respectively, and use the PBS solution at the same pH value as a control. Carry out bacteriostasis experiments and measure the diameter of the bacteriostatic zone. The relative remaining antibacterial activity is the ratio of the diameter of the inhibition zone of the experimental group to the diameter of the control group, which is used to analyze the influence of pH on the antibacterial activity.

### The Effect of Lactic Acid on Antibacterial Activity

The CFS of *L. plantarum* KLDS 1.0344 fermentation to different time points (8 hours, 16 hours and 24 hours) was collected. The lactic acid content of the CFS at 8 h, 16 h, and 24 h was measured by high-performance liquid chromatography (21). Perform antibacterial experiments on CFS at different time points and measure the diameter of inhibition zone. The data between the diameter of inhibition zone and the lactic acid content were fitted and analyzed to investigate its effect on the inhibition activity.

### Induction of Inflammatory Response in bMEC

The bMEC were seeded in a 6-well plate at a 10^5^ cells/well rate to induce mastitis experiments. The L, M, and H groups were adding different concentrations of *L. plantarum* KLDS 1.0344 (1.0×10^5^, 10^6^, 10^7^ cfu/well) to the wells of the cultured cells and co-cultured with the cells for 1 h. Then 1 μg/mL LPS was added to L, M, H, and LPS (model control group) groups to induce inflammation. The NC group served as the control group and only added a high-sugar DMEM medium containing 10% fetal bovine serum. After 18 h of treatment of bMEC with *L. Plantarum* and LPS, the bMEC were washed with PBS twice, and harvested for RNA extraction. RNA extraction steps for the bMEC were performed according to the RNA extraction kit instructions (Tiangen Biotech Co., LTD., China). The primers were obtained on National Centre for Biotechnology Information (NCBI), designed with premier software, and synthesized as shown in **Table S1**. Analysis was performed using the qRT-PCR technique previously reported with minor modifications (22, 23). cDNA was obtained by reverse transcription using PrimeScript RT kit with gDNA Eraser (Takara, Japan). SYBR Premix Ex Taq (Takara, Japan) was used for mRNA expression of genes through the ABI 7500 fast real-time PCR system (Applied Biosystems, USA). The mRNA expression levels of TNF-α, IL-6, and IL-1β in breast epithelial cells were detected.

### Animals and Experimental Design

A total of 40 pregnant BALB/c mice were provided by the Laboratory Animal Center of Harbin Medical University (Harbin, China) and housed in a room under controlled environmental conditions at 20~25°C and 12h light/dark cycle. The mice were placed in plastic cages for five days to acclimatize before the experiments and provided a standard chow diet and water *ad libitum*. All mice received human care, and all of the animal procedures presented in this experiment were performed following the guidelines of the Northeast Agricultural University for the use of laboratory animals; all experiments were reviewed and approved by the Northeast Agricultural University.

They were randomly assigned to 4 groups (n=10): normal control group (NC), model control group (MC), positive control group (PC), and *L. plantarum* KLDS 1.0344 dose group. Firstly, the NC group and MC group were intragastrically administered with 0.25mL PBS. The PC group was intravenously injected with the same amount of 5 mg/kg dexamethasone. The *L. plantarum* KLDS 1.0344 group was given an equal amount of 5.0×10^9^ CFU/mL live bacteria suspension by gavage. Then let the NC group eat freely, and the other three groups were all constructed mice mastitis models. The mouse model of LPS-induced mastitis was established by injection of LPS, as described by Shao (24), Briefly, lactating mice were anesthetized by ethyl ether, and the very near ends of the fourth pair of breast glands in the abdomen were cut. 50 µL LPS (0.2 μg/μL) was infused into the mammary gland through the duct of the mammary gland. The control mice were infused with an equal amount of sterile PBS into the mammary gland through the duct of the mammary gland. An inflammation model was established one hour after LPS treatment. At 24 h after the model was established, the mice were humanely sacrificed with CO_2_ inhalation, and then the fourth pair of mammary tissues were collected and stored at -80°C.

### Histopathologic Analysis

Histopathologic analysis was performed as described in the previous research (25). Briefly, we fixed the samples in 4%-buffered paraformaldehyde solution, dehydrated them in ethanol, embedded them in paraffin, and cut them into 4 μm sections. After staining with standard hematoxylin-eosin (HE), the pathological changes of breast tissue after different treatments were observed under light microscope. The ×400 magnification was selected to observe breast tissue samples.

### Determination of MPO Activity and Cytokine Levels

The mouse breast tissue and PBS were transferred into a glass homogenizer, and thoroughly grind on ice. The solution was centrifuged at 5000 r/min for 5 minutes to obtain the supernatant for detection. the MPO activity level in breast tissues was detected using the MPO kit. (Nanjing Jiancheng Bioengineering Institute, Nanjing, China). We used the enzyme­linked immunosorbent (ELISA) kits (Nanjing Jiancheng Bioengineering Institute, Nanjing, China) to measure the levels of TNF­α, IL­1β, IL­6, in breast tissues.

### Western Blotting Detects the Expression of Signal Pathway Proteins

The total proteins were extracted from mouse breast tissue. The protein concentration was determined by the BCA protein assay kit (Tiangen Biotech, China). 20 μg of the protein samples per lane were subjected to SDS-PAGE and then transferred to polyvinylidene difluoride (PVDF) membranes. The PVDF membrane was transferred to the blocking solution and sealed on a shaker with slow shaking for 1 h, followed by incubation with primary antibodies that recognized p65, p-p38 (1:500; ImmunoWay, USA), IκB, p-IκB, p- β-actin (1:1,0000; Abcam, UK), COX2 (1:2000; Abcam, UK), iNOS (1:1000, Sangon Biotech, China) at 4°C overnight. These were then incubated with the secondary antibody at 4°C in the dark for 2 hours. Finally, the ECL chemiluminescence reagent was used for color rendering. Use Gel-Pro-Analyzer to measure and analyze the optical density value of the target band.

### Statistical Analysis

For this study, the SPSS 22.0 was applied for data processing, and the *post hoc* comparison tests (Duncan’s Multiple Range Test, DMRT) was used to compare the significance of differences between groups. A *P* value < 0.05 indicates significant differences, and the Origin software (v9.0) was used for plot analysis.

## Results

### Bactericidal Properties of *L. plantarum* KLDS 1.0344

**Figure 1:**
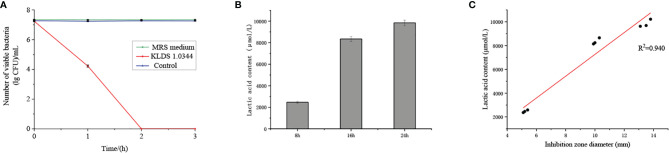
**(A)**. The bactericidal effect of CFS on *Escherichia coli* ATCC25922 was quantified by the lgCFU value of the number of viable bacteria. The control group was treated with saline, and the MRS medium without bacteria was adjusted to pH = 4. **(B)** The contact dependence inhibition effect was expressed by the number of viable bacteria under the co-cultivation of *Escherichia coli* ATCC25922 and *L. plantarum* KLDS 1.0344, and the control group is *Escherichia coli* ATCC25922 cultured alone. **(C)** Lactic acid content in the cell-free fermentation supernatant at different fermentation times. **(D)** Linear analysis of lactic acid content and bacteriostatic circle diameter. The values obtained for all tests are the mean ± SD (n = 3 independent experiments).

The bactericidal effect of CFS on *E. coli* ATCC25922 was shown in **Figure 1A**. In the case of co-cultivation of *E. coli* and *L. plantarum*, the number of viable bacteria decreased rapidly with time, and no viable bacteria existed within 2 hours. The number of viable bacteria in the control group (treated with saline) hardly changed over time. The number of viable bacteria was not affected under the treatment of MRS medium with pH equal to CFS. Therefore, the CFS has a bactericidal effect on *E. coli.*

The effect of NaOH at pH 6.5, proteinase K, catalase, pepsin, and trypsin on the antibacterial activity of CFS was shown in **Table 1**. No bacteriostatic activities of CFS under NaOH treatment at pH 6.5. There is no significant change in CFS’s remaining bacteriostatic activities after proteinase K, papain, and trypsin treatment. This tentatively suggests that *L. plantarum* KLDS1.0344 may not produce protein substances such as bacteriocins to kill the pathogen strain of *E. coli*. The genes related to bacteriocin production by *Lactobacillus plantarum* were amplified, and no related genes were amplified using this strain as a template, which further indicated that the bacteriocin-related proteins are not produced by *L. plantarum* KLDS 1.0344. Similarly, after adding an appropriate amount of catalase (1 mg/mL) to the supernatant of *Lactobacillus*, the antibacterial activity was unchanged, indicating that the antibacterial activity was not exerted by catalase. With the increase of pH, the antibacterial activity of CFS is affected (**Table 2**), indicating that the external acidic conditions can hinder the antibacterial activity. At the same time, no antibacterial activity was shown in PBS (pH=4) treatment. The results show that the antibacterial activity of the substances in CFS under low pH conditions is the main reason. This phenomenon might be related to organic acids produced by *L. plantarum* KLDS 1.0344.

**Table 1:** Effects of catalase, protease treatment, pH, and Lactic acid on the bacteriostatic activity of the CFS derived from *L. plantarum* KLDS 1.0344.

Treatment	Remaining bacteriostatic activity (%)
pH 6.5	0 ± 0
1 mg/mL Catalase	96.54 ± 0.54
1 mg/mL proteinase K	93.71 ± 0.54
1 mg/mL papain	95.60 ± 0.55
1 mg/mL trypsin	98.43 ± 0.55
Lactic acid	97.80 ± 1.44

Remaining bacteriostatic activity of CFS with different treatments was presented as percentages (%). Remaining antibacterial activity was expressed as the ratio of the experimental group’s inhibition zone’s diameter and the diameter of the control group. Values are mean ± SD (n = 3 independent experiment).

**Table 2:** Bacteriostatic activity of the CFS against *E. coli* ATCC 25922 by different pH treatments.

Treatment	Remaining bacteriostatic activity (%)
PBS (pH 4.0)	0 ± 0
Untreated CFS	100 ± 0
pH 4.5	50.32 ± 0.04
pH 5.0	22.08 ± 0.05
pH 5.5	0 ± 0
pH 6.0	0 ± 0

Values are mean ± SD (n = 3 independent experiment). Remaining bacteriostatic activity of CFS with different treatments was presented as percentages (%). Remaining antibacterial activity was expressed as the ratio of the experimental group’s inhibition zone’s diameter and the diameter of the control group.

Lactic acid treatment and CFS showed similar antibacterial properties (**Table 2**), so we further tested the lactic acid content at different time points (**Figure 1B** **)**. At the 8 h time point, the lactic acid content in CFS of *L. plantarum* KLDS 1.0344 was 2471.01 ± 89.91 µmol/L, while at the 16 h and 24 h time points, the lactic acid content was 8343.52 ± 221.04 µmol/L and 9842.06 ± 267.08 µmol/L, respectively. With the increase of time, the lactic acid content also gradually increased. There is a positive correlation between the lactic acid content and the diameter of the inhibition zone (**Figure 1C** **)**. The correlation coefficient is R=0.940, and the bacteriostatic effect gradually increases as the lactic acid content increases. The results show that lactic acid is the main antibacterial substance in CFS of *L. plantarum* KLDS 1.0344.

### Effect of *Lactobacillus plantarum* KLDS 1.0344 on the Immune Activity of bMEC

**Figure 2:**
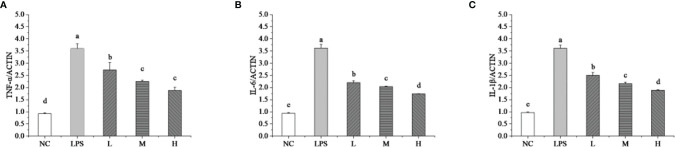
Effect of *L. plantarum* KLDS 1.0344 on the expression levels of cytokine genes in bMEC. **(A)** TNF-α; **(B)** IL-6; and **(C)** IL-1β. NC represents unstimulated bMEC; LPS represents LPS-stimulated bMEC; L, M, and H represent LPS-stimulated bMEC co-incubated with 10^5^, 10^6,^ and 10^7^ CFU/mL *L. plantarum* KLDS 1.0344, respectively. Values are mean ± SD. Means with no common letters differ significantly (P < 0.05).

The RT-qPCR technique detected the mRNA expressions levels of TNF-α, IL-6, and IL-1β to evaluate the effects of *L. plantarum* KLDS 1.0344 on LPS-induced mastitis using isolated bMEC. As shown in **Figure 2**, compared with the NC group, the stimulation of bMEC with LPS led to an up-regulation of all the pro-inflammatory genes (p < 0.05). Compared with the NC group, the LPS group can significantly increase the relative expression levels of the TNF-α gene (p < 0.05). After stimulating bMEC with a low dose of *L. plantarum* KLDS1.0344, it can reduce the TNF-α gene expression levels (p < 0.05). After stimulating the cells with the ratio of medium and high doses, although it can continue to reduce the expression levels of the TNF-α gene (p < 0.05), there is no significant difference between the two (*P* > 0.05) and there is no dose-dependent relationship. Similarly, compared with the NC group, the LPS group can also significantly increase the relative expression levels of the IL-6 and IL-1β genes (p < 0.05). After treating the *L. plantarum* KLDS1.0344 at different concentrations to stimulate bMEC, it can significantly reduce the IL-6 gene levels. For the level of the IL-1β gene, we observed the same changes as the expression levels of the IL-6 gene. In this study, the results indicate that *L. plantarum* KLDS1.0344 can play a probiotic function to alleviate inflammation by regulating the expression levels of TNF-α, IL-6, and IL-1β mRNA in bMEC.

### HE Pathological Changes of Breast Epithelial Cells

**Figure 3:**
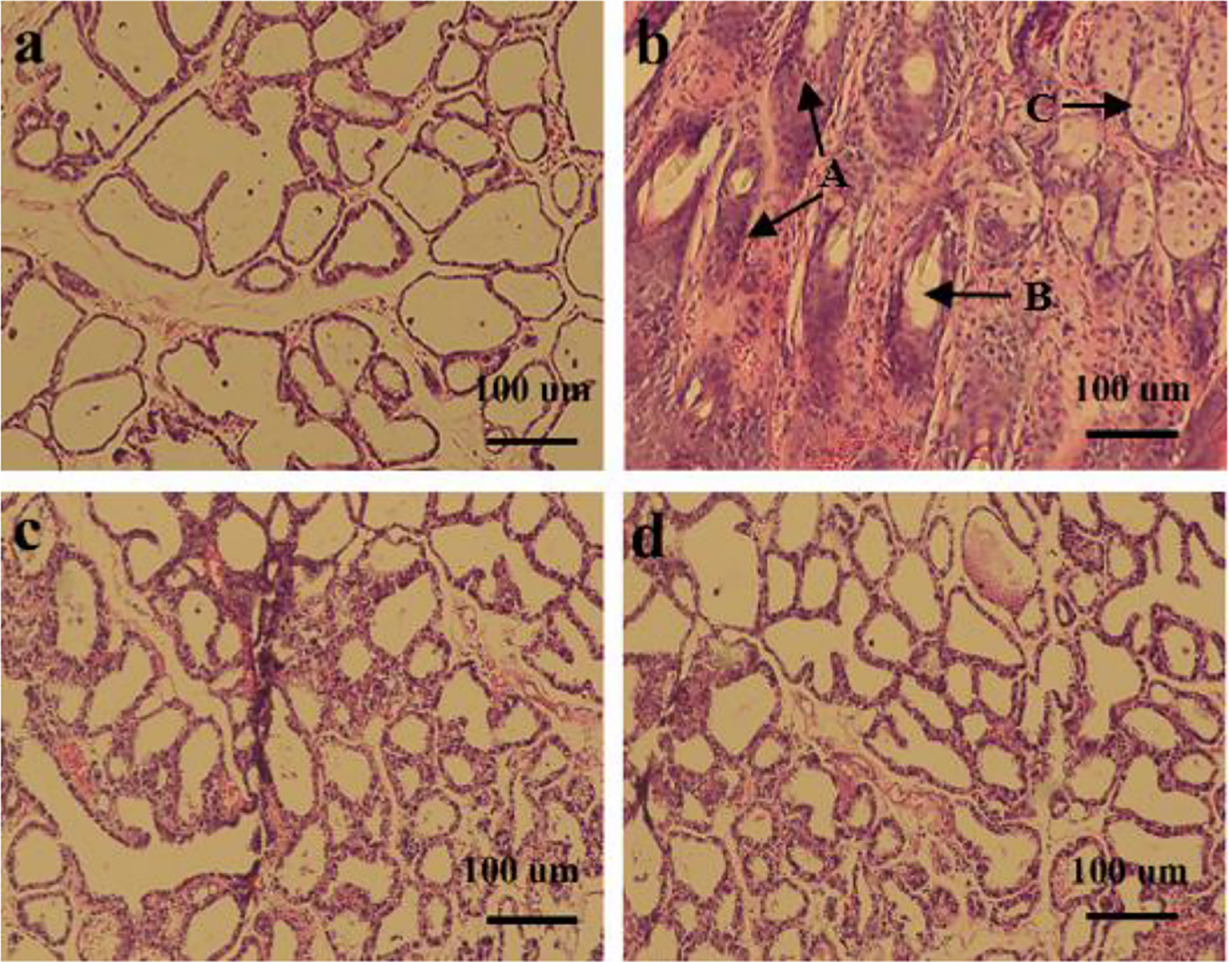
Histopathological changes of mouse mammary glands **(A)** control group; **(B)** LPS group; **(C)** *Lactobacillus plantarum* pretreatment group; **(D)** dexamethasone group. The picture indicates A = Tissues necrosis (degeneration), B = Polymorphonuclear neutrophil (PMN) and lymphocytes, C = macrophages.

The effect of *L. plantarum* KLDS 1.0344 on LPS-induced mastitis in lactating mice was studied. Mice infected with LPS gradually developed clinical mastitis (painful, red, and swollen breasts) with the onset of lethargy. The pathological analysis of breast epithelial cells under the microscope is shown in **Figure 3**. Infected mice developed severe acute mastitis (Group b). Mice’s mammary epithelial lobules, interstitial fibrovascular tissue, and interlobular adipose tissue infiltrated a mixed group of inflammatory cells, including lymphocytes and plasma cells, neutrophils, macrophages (26). Group a is a blank control group; the mouse mammary epithelial cell acinar structure is complete without noticeable pathological changes. Group c is the *L. plantarum* KLDS 1.0344 pretreatment group, the *L. plantarum* KLDS 1.0344 pretreatment inhibits the tissue damage of mice mammary epithelial cells caused by LPS to a certain extent. Meanwhile, the exudation of neutrophils in the breast epithelial cell tissue is reduced, and the tissue congestion is weakened. Group d was the dexamethasone positive control group, and the dexamethasone treatment group could significantly inhibit breast tissue damage caused by LPS.

### Myeloperoxidase Activity Detection

**Figure 4:**
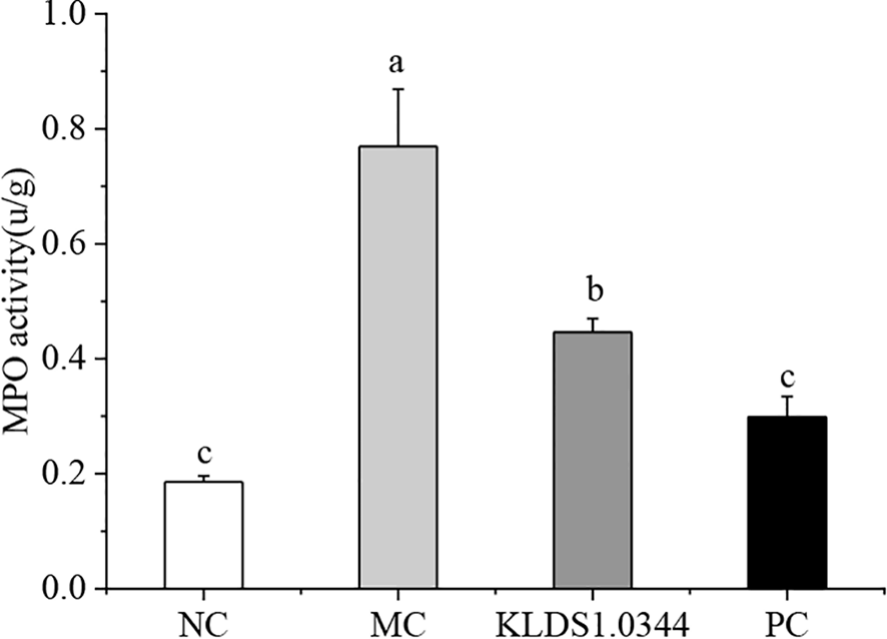
Effect of *Lactobacillus plantarum* KLDS 1.0344 treatment on MPO activity in breast tissue. NC, normal control group; MC, LPS-induced mastitis group; KLDS1.0344, KLDS1.0344 pretreatment group, PC, pretreated with dexamethasone group. Values are mean ± SD. Means with no common letters differ significantly (P < 0.05).

MPO activity is a typical marker of inflammatory cell penetration. This experiment measured MPO activity in mice mammary tissues. The results in **Figure 4** show that the activity of MPO in the LPS model control group (MC) was significantly enhanced (*P* < 0.05). Pretreatment with *L. plantarum* KLDS 1.0344 to a certain extent reduced the MPO activity after LPS stimulation (p <0.05). In the positive control group (PC), dexamethasone pretreatment significantly reduced MPO activity.

### Detect the Levels of Inflammatory Factors IL-6, IL-1β, and TNF-α

**Figure 5:**
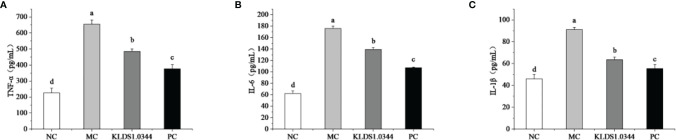
Effects of pretreatment of KLDS 1.0344 on the expression levels of IL-6 **(A)**, TNF-α **(B)**, and IL-1β **(C)** in breast tissue. NC, normal control group; MC, LPS-induced mastitis group; KLDS1.0344, KLDS1.0344 pretreatment group, PC, pretreated with dexamethasone group. Values are mean ± SD. Means with no common letters differ significantly (P < 0.05).

The levels of inflammatory factors in breast tissue measured by ELISA kit are shown in **Figure 5**. Compared with the NC group, the expression levels of IL-6 in the MC group were significantly increased (*P* < 0.05), and after pretreatment with *L. plantarum*, the expression levels of IL-6 were reduced to a certain extent. The levels of IL-6 in the dexamethasone positive control group (PC) were also significantly lower than that in the MC group, and there was also a significant difference between the PC group and the NC group (*P* < 0.05).

Compared with the NC group, the expression levels of the IL-1β in MC increased significantly (*P* < 0.05), while after pretreatment with *L. plantarum*, the expression levels of the IL-1β decreased. The levels of 1β were also significantly lower than that of the MC group. Furthermore, compared with the NC group, the expression of TNF-α in the MC group was significantly increased (*P* < 0.05), and after pretreatment with *L. plantarum*, the expression levels of TNF-α were reduced. The levels of TNF-α in the PC group were also significantly lower than those in the MC group.

### Protein Expression of the Signaling Pathway in Breast Tissue

**Figure 6:**
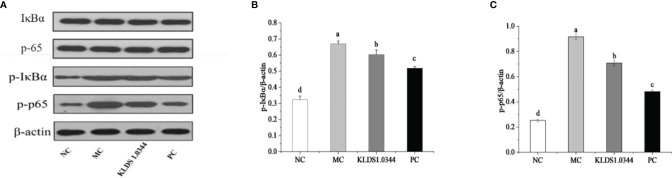
The western blot detection map **(A)** of the tested strains affecting the NF-κB signaling pathway. LPS-induced expression of p-IκBa **(B)** and p-p65 **(C)** protein in the NF-κB pathway of mouse breast tissue. NC, normal control group; MC, LPS-induced mastitis group; KLDS1.0344, KLDS1.0344 pretreatment group, PC, pretreated with dexamethasone group. Values are mean ± SD. Means with no common letters differ significantly (P < 0.05).

Detection of NF-κB signaling pathway protein expression by Western blotting is shown in **Figure 6**. The NF-κB signaling pathway is essential in producing inflammatory cytokines, the phosphorylation level of NF-κB signaling pathway protein. The test results showed that compared with the NC group, the phosphorylation levels of p65 and IκBa in the MC group were significantly increased after LPS stimulation (*P* < 0.05). The phosphorylation levels were significantly reduced in the *L. plantarum* KLDS1.0344 pretreatment group and the PC group. The expression levels of p-IκB and p-p65 protein induced by LPS stimulation (p < 0.05). The results showed that pretreatment with *L. plantarum* KLDS1.0344 could reduce the phosphorylated expression levels of p65 and IκB in the NF-κB signaling pathway, thereby controlling the expression of downstream inflammatory factors.

## Discussion

Cow mastitis is the most severe disease causing economic losses in dairy farming (27), which is a subject of continuous attention and research by scholars at home and abroad (28, 29). So far, antibiotics are still the primary treatment for mastitis in cows. However, the heavy use of antibiotics can increase strain resistance, resulting in difficulty curing mastitis (10, 30). Therefore, finding an alternative to antibiotics is a priority in research efforts against mastitis in dairy cows.

*L. plantarum* has been continuously studied as a new effective substance against mastitis. It plays a role by competitively inhibiting pathogenic bacteria or producing metabolites through fermentation. The metabolites of *L. plantarum* have been widely verified and considered antibacterial activity, mainly organic acids, bacteriocins, and hydrogen peroxide (14). In some recent studies, some *Lactobacillus* strains were found to be able to inhibit a variety of mastitis pathogenic bacteria such as *E. coli*, and was able to avoid the emergence of drug-resistant strains, which was considered as a possible alternative to antibiotics (31–34). Bacterial inhibition performance was used as a critical indicator for the initial screening of superior strains. Based on previous reports and research methods, *L. plantarum* KLDS1.0344 was selected as the subject of subsequent experimental research. After co-cultivating *E. coli* ATCC25922 and CFS for 1 hour, *E. coli* partially survived, but no *E. coli* survived for 2 hours after co-cultivation. It indicates that the metabolites secreted by the fermentation of lactic acid bacteria can inhibit pathogenic bacteria.

As mentioned above, the antibacterial effect of lactic acid bacteria is mainly attributed to some antibacterial substances produced in the metabolic process, such as hydrogen peroxide, bacteriocins, and organic acids. In this study, the antibacterial activity was unchanged after treatment with catalase, indicating that the antibacterial substance has nothing to do with catalase. It is not sensitive after being treated with protease substances, and the antibacterial effect may not be related to protease substances. At the same time, the bacteriocin-related genes were amplified, and the expression of related genes was not detected. Further research on the correlation between pH and antibacterial activity found that as the pH value gradually increases, the antibacterial activity gradually decreases. It shows that the substances with antibacterial activity in the metabolites of lactic acid bacteria are indeed organic acids. By measuring the correlation between lactic acid and bacteriostatic activity, the content of lactic acid and the diameter of the bacteriostatic zone showed an apparent positive correlation, with a correlation coefficient of more than 90%, which indicated that the lactic acid in organic acids played a significant role in inhibiting *E. coli* ATCC25922.

The inflammatory factors TNF-α, IL-6, and IL-1β, are closely associated with the response to bacterial infection (35–38). Some reports such as LPS can stimulate inflammation in animal mammary epithelial cell models (39, 40). LPS leads to the production of inflammatory cytokines such as TNF-a mainly through macrophages and monocytes’ stimulation (41, 42). LPS is an important promoter in the NF-κB inflammatory signaling pathway (43, 44). The ability to stimulate TLR4 expression on the cell surface resulted in significant upregulation of both NF-κB and MAPK signaling pathways downstream. In this study, treatment with *L. plantarum* KLDS1.0344 reduced mRNA expression for TNF-α, IL-6, and IL-1β, and. This indicates that LPS-induced inflammation is alleviated to a certain extent. Previous studies have shown that these inflammatory cytokines were regulated by the NF-κB signaling pathway (45). Therefore, a mouse model of mastitis was established to further study the mechanism of *L. plantarum* KLDS1.0344 affecting mastitis *in vivo*.

As an important mediator of inflammation, LPS has been widely used to establish mastitis models in mouse (24). The mouse mastitis model established by injection of LPS was consistent with the clinical symptoms of cow mastitis (46). This experiment studied the preventive and protective effects of *L. plantarum* KLDS 1.0344 through an animal model of LPS-induced mastitis in mice. The results showed that the acinar structure of the breast tissue of mice in the LPS model group (MC group) changed, resulting in the infiltration of inflammatory cells. In addition, the content of inflammatory cytokines such as TNF-α was significantly increased. The pretreatment of *L. plantarum* KLDS 1.0344 significantly improved this situation. MPO activity is a typical sign of inflammatory cell infiltration (47), so it is widely used to assess the condition of mastitis (48). LPS stimulation leads to a significant increase in MPO activity, which means that neutrophils accumulate at the site of infection. *L. plantarum* KLDS 1.0344 pretreatment and dexamethasone can significantly reduce MPO activity to a certain extent.

LPS is an important substance that initiates the NF-κB inflammation signaling pathway. The transcription of the initiating gene induces the production of inflammatory-related enzymes and other substances, thereby regulating the expression of inflammatory factors. These genes play vital roles in innate and adaptive immune regulation, cell adhesion, inflammatory response, and anti-apoptotic mechanisms (49). In the MC group, the expression of p-p65 and p-IκBa proteins of the nuclear transcription factor NF-κB signaling pathway was significantly increased, indicating that a mouse mastitis model had been established. Both indices were significantly reduced after treatment with *L. plantarum* KLDS1.0344. *L. plantarum* KLDS1.0344 can regulate the activation of the NF-κB signaling pathway to prevent breast inflammation. The results suggest that supplementation with *L. plantarum* KLDS1.0344 can effectively restore the physiological changes in mice treated with LPS.

## Conclusion

In summary, *in vitro* and *in vivo* results from our study demonstrated that *L. plantarum* KLDS1.0344 has the effect of preventing and protecting mastitis. *L. plantarum* KLDS1.0344 inhibits pathogenic *E. coli* through fermentation to produce lactic acid. In addition, *L. plantarum* KLDS1.0344 regulates the NF-κB signal activation pathway, thereby reducing the levels of related inflammatory cytokines in mastitis.

## Data Availability Statement

The datasets presented in this study can be found in online repositories. The names of the repository/repositories and accession number(s) can be found in the article/**Supplementary Material**.

## Ethics Statement

The animal study was reviewed and approved by Northeast Agricultural University.

## Author Contributions

QC and SW contributed to conception and design of the study. QC organized the database. QC performed the statistical analysis. JG wrote the first draft of the manuscript. QX, SE, BL and GH wrote sections of the manuscript. YS revising the content and interpretation of data. All authors contributed to manuscript revision, read, and proved the submitted version.

## Funding

This study was financially supported by “Hundred, Thousand and Ten Thousand” Science and Technology Major Special Project of Heilongjiang Province: Dairy Products and Meat Processing (No. 2020ZX07B01), the Natural Science Foundation of Heilongjiang Province (YQ2020C013), the National Natural Science Youth Foundation of China (No. 31801518), Chinese nutrition society - Feihe physique nutrition and health research fund (CNS-Feihe2020A37), Academic Backbone Plan of Northeast Agricultural University (No. 19YJXG10), and the “Young Talents” Project of Northeast Agricultural University (18QC52).

## Conflict of Interest

Author QX is employed by Heilongjiang Feihe Dairy Co., Ltd.

The remaining authors declare that the research was conducted in the absence of any commercial or financial relationships that could be construed as a potential conflict of interest.

## Publisher’s Note

All claims expressed in this article are solely those of the authors and do not necessarily represent those of their affiliated organizations, or those of the publisher, the editors and the reviewers. Any product that may be evaluated in this article, or claim that may be made by its manufacturer, is not guaranteed or endorsed by the publisher.

## Supplementary Material

Supplementary Figure 1 Key gene sequence related to bacteriocin synthesis of *Lactobacillus plantarum*.

Supplementary Table 1 Primer sequences for reverse transcription PCR.
