# Supplementary material for: The Protective Effects of Lactobacillus plantarum KLDS 1.0344 on LPS-Induced Mastitis In Vitro and In Vivo
Source: Front Immunol. 2021 Nov 9;12:770822. doi: 10.3389/fimmu.2021.770822 (PMC8630701; doi:10.3389/fimmu.2021.770822)
Supplement: Supplementary Figure 1 — Key gene sequence related to bacteriocin synthesis of Lactobacillus plantarum. [file DataSheet_1.pdf]

Table S1 Primer sequences for reverse transcription PCR

gene	Primer direction Size (bp)	Sequence (5'-3')	(bp)
IL-1β	Forward	ATGAAGAGCTGCATCCAACACCTG	110
	Reverse	ACCGACACCACCTGCCTGAAG	
IL-6	Forward	GCCTTCACTCCATTCGCTGTCTC	117
	Reverse	AAGTAGTCTGCCTGGGGTGGTG	
TNF-α	Forward	CTGGCGGAGGTGCTCTC	85
	Reverse	GGAGGAAGGAGAAGAGGCTGAGG	
ACTIN	Forward	ATGTGGAGCTGGCGGAGGAG	315
	Reverse	CGAGGGCATTGGCATACGAGTC	

（1）PlnC8A
ATGGATAAATTTGAAAAAATTAGTACATCTAACCTAGAAAAGATCTCTGGCGGTGA
AACAACCAAGTTATGGAGCTCTTGGGGATATTATCTTGGCAAGAAAGCACGTTGGAA
TAAAGCACCCATATGTTCAATTTTAG
（2）PlnC8IF
ATGAAAAACATTAATAAGTACACTGAACTAAATGATCAGAAGTTGCAATCTTTAATT
CGGAAAAACAAAGACTATAAGCTTGATGTCGGGGTTGCAACTCCCCCATGCTTTTACA
AAATTATTAAAAGCACTTGGAGGCCATCATTAG
（3）PlnC
GTGTTTCCAATTTATTTATTAGAAGATAACGAGGCCCAAAGAGTAGAGTATATAAGT
ATAAAGAATATAATCATGATTAAAGAATATGATATGCAGTTGGTGGTGGCGACAGG
TTTACAAGAGTTAATGAATAATGTTATGAATTCTAAAGAAGGGCTGTTTTTCTTGGAT
GGAAATTGGGGAACAAACTCAGGCCGGGTTGAATTTAGCAGATGAAATTCGGCAGCA
GTTACCTTGTGCACAAATTGTATTTATTACAACTCATGAAGAGTTATCTTTTTTGACA
GGAGCGGCGGATTGCACCGTTGGATTATATTTTGAAAGAACAAGGCCTTGATGATA
AGCAAAAAATAGTTAAGGATATTGATGCAACTCAAACTATTCTCAAGACAGAAACTG
GCAGCATAAGGATATTTTAGGTTATAAAATCGGAACGCGTTCTTTTCAGTTCCTATTAA
TGATGTTATTATGCTGAGCACTAATAAAGAACGACCGGGTAGTATTAGGCTGACTGC
AAAATAAAGTGGCAGATTTTCCAGGTAATTTAAATAGTTTTGAAAATAAATACTCAC
TTTTTTAGGTGTGATAAAAGTTCATTGGTAAATATAGATTATGTTGATAGTTATGATT
AAAAAAAAGAGCTTACCATGATAGATAACATTAAGTGCAGTGTTTCGTATAGAAAGTT
CGGGAGCTTAACAAAATATTGAAAAAGAAATAG
（4）PlnD
TTGTTTCCAATTTATTTATACGAGGATAATGCTGAGCAAAGAGATAATTATTGTAAGA
GTCAATAATACAATTATGATTAATGAGTTTGCAATGGAGCTTAGGGTTGCAACGGAT
CAAAAAATAATTCTAGCAGATTTGAATCAACAACAAGATGGCCTTTTCTTTTTGGATA
GGAAATTGGTGAGGACAAACAGACTGGACTTGAATTAGCCAGTCGAATTCGGGCAAC
GATACCATTGGCTAAAATAGTTTTCATTACAACACACGATGAGCTATCGTTTGTAACT
GGAACGGCGGATTGCACCGTTGGATTATATTTTGAAAGACCAGTCTGCTGACCTAAT
CGCAAAGGATTATTAAGGACATCAATGTAGTACAGAACGAATTAAAAAAGACTAAT
CAGCGC
AAAGATGTTTTTAACTATAAGTTAGGAACGCGATACTTTTCACTGCATTAGATGATGT
ATTTTGTTGAGTACATCTAAACTGCGTCCGGGCAGCGTACAACTCCATGCTATTAATA
GGTTGCTGAGTTCCCAGGAAATTTAAATGCGCTCGAAGAAAAGTATCCGCAATTTTT
GATGCGATAAGAGCTCGCTGGTAAATTTAAATCATTTGCGAAGTTTTGACTATAAAG
AAAGAGTTGTTGCTCGACGGTGAAATTAGGTGTAAGGCTTCGTTTAGAAAGTCGCGG
AATTGAA
（5）PlnE
ATGCTACAGTTTGAGAAGTTACAATATTCCAGGTTGCCGCAAAAAAAGCTTGCCAAA
TATCTGGTGGTTTTAATCGGGGCGGTTATAACTTTGGTAAAAGTGTTCGACATGTTGT
ATGCAATTGGTTCAGTTGCAGGCATTCGTGGTATTTTGAAAAGTATTCGTTAA
（6）PlnK
ATGAAAATTAAATTAACTGTTTTAAATGAATTTGAAGAATTAACTGCTGACGCTGAA
GAATATTTCTGGTGGCCGTCGGAGTCGTAAAAATGGAATTGGATACGCTATTGGTTAT
CGTTTGGCGCGGTTGAACGGGCCGTGCTTGGTGGTTCAAGGGATTATAATAAGTGA

Figure S1 Key gene sequence related to bacteriocin synthesis of Lactobacillus plantarum
